# Editorial: Highlights of ENPER 2019—European Network for Plant Endomembrane Research Meeting

**DOI:** 10.3389/fpls.2021.719367

**Published:** 2021-07-09

**Authors:** Fernando Aniento, Erika Isono, Enrique Rojo, Eugenia Russinova

**Affiliations:** ^1^Departamento de Bioquímica y Biología Molecular, Instituto Universitario de Biotecnología i Biomedicina (BIOTECMED), Universitat de València, Valencia, Spain; ^2^Department of Biology, University of Konstanz, Konstanz, Germany; ^3^Centro Nacional de Biotecnología, Consejo Superior de Investigaciones Científicas, Madrid, Spain; ^4^Department of Plant Biotechnology and Bioinformatics, Ghent University, Ghent, Belgium; ^5^Center for Plant Systems Biology, Vlaams Instituut voor Biotechnologie, Ghent, Belgium

**Keywords:** plant endomembranes, ER-golgi trafficking, autophagy, endocytosis, exocytosis, vacuolar trafficking

ENPER (European Network for Plant Endomembrane Research) meeting is the first and oldest international conference of the plant membrane research community specifically devoted to studying plant membrane functions. The ENPER was created in 1996 by Professor David Robinson (University of Heidelberg, Germany), and since then it has been organized every year in different countries, bringing together many different groups working on plant endomembranes from Europe and around the world. A strong emphasis has always been made at the ENPER meetings to give young researchers (PhD students and Post-docs) the opportunity to present their work in front of a highly specialized audience, and this was also the spirit in the last meeting, right before COVID-19 pandemics, which took place in the beautiful city of Valencia (Spain) (Figure 1).

**Figure 1:**
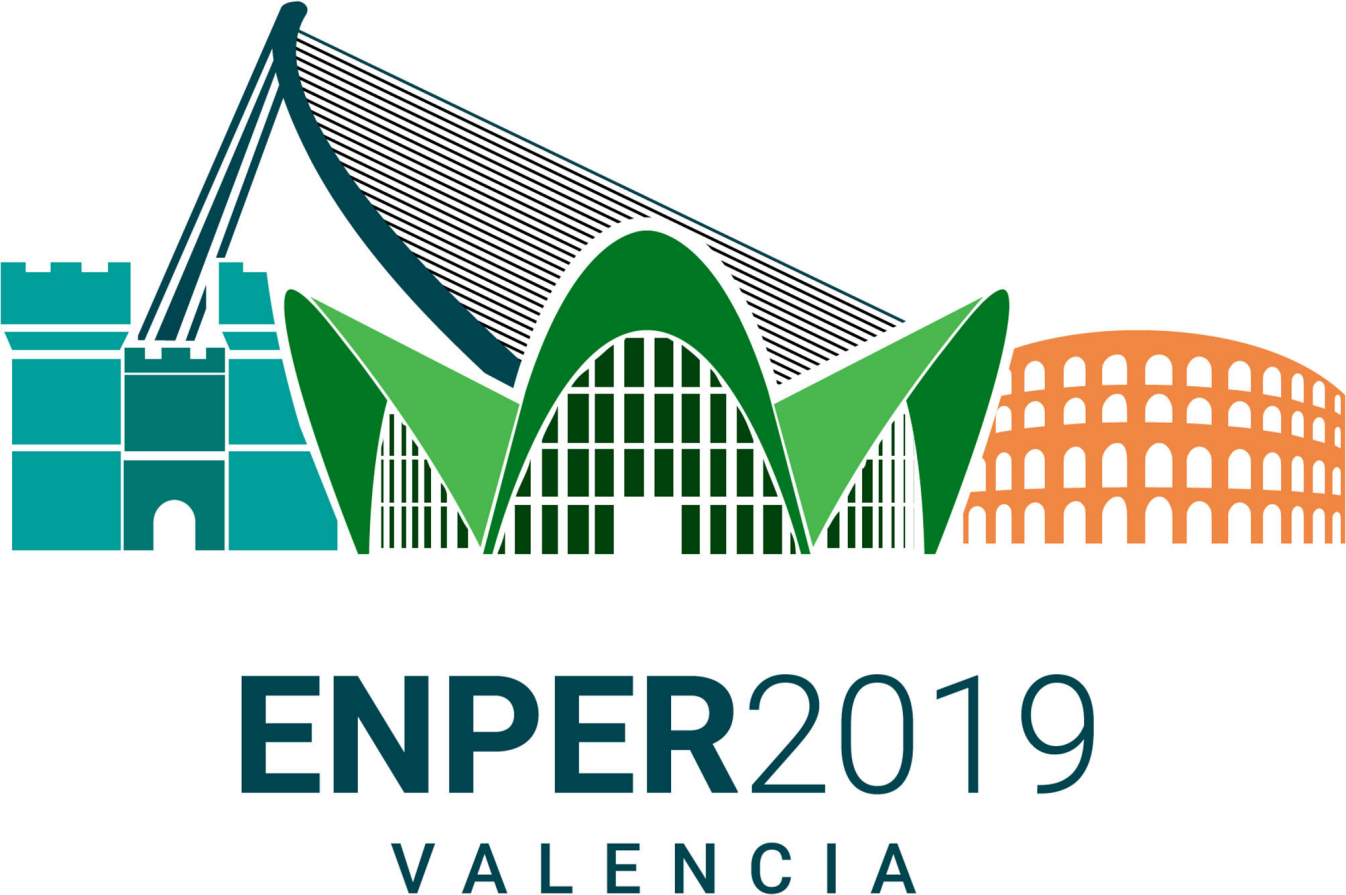
Logo of ENPER 2019.

The program of the meeting included sessions on different aspects of plant endomembrane trafficking, including “Autophagy,” “Endoplasmic reticulum and ER-Golgi trafficking,” “Golgi complex and *trans*-Golgi network,” “Endocytosis and endosomal transport,” “Trafficking and polarity,” “Exocytosis and defense,” and “Vacuolar trafficking.” This Research Topic includes articles from several participants in the meeting and covers different aspects of plant endomembrane trafficking.

Ito and Boutté discuss the subdomain organization of the plant Golgi apparatus focusing on the Golgi entry and exit compartments, the Golgi Entry Core Compartment (GECCO) and the *trans*-Golgi network (TGN), respectively. Both subdomains define distinct protein sorting mechanisms and trafficking pathways. The GECCO may be the plant counterpart of the mammalian endoplasmic reticulum (ER)-Golgi Intermediate Compartments (ERGIC), but in plants, ER-to-GECCO transport is independent from COPII machinery. Whereas, TGN subdomains have been characterized to some extent, the GECCO vesicles have not been isolated yet. GECCO and the first *cis*-Golgi cisternae might be specialized in cargo sorting. Live-cell imaging have demonstrated that TGN can either be associated with the *trans* side of the Golgi apparatus (Golgi-associated TGN/GA-TGN), or can be disassociated and move independently from the Golgi (Golgi-independent TGN/GI-TGN or free TGN). Once formed, TGN is able to further differentiate into other compartments of different composition. The TGN is known to not only receive secretory cargos from the Golgi but also cargos from the endocytic pathway and it is assumed that the TGN is equivalent to the early endosomes (EEs) in plant cells. Trafficking from TGN to late endosomes/multivesicular bodies is thought to partly rely on clathrin-coated vesicles (CCVs) and the SNARE VAMP727. At clathrin/TGN subdomain, the small GTPase RAB-A2a is involved in endocytic sorting to the plasma membrane through the action of the sphingolipid ceramides whereas at secretory vesicles/TGN, the ECH/YIP4 complex is involved in secretory sorting to the plasma membrane.

Sanchez-Simarro et al. analyzed the function of the two isoforms of beta-COP, one of the subunits of the Coat Protein I (COPI) coatomer complex, involved in the formation of vesicles at the Golgi apparatus, for intra Golgi transport or Golgi-to-ER retrograde transport. Using a loss-of-function approach, the authors show that β-COP is required for plant growth and tolerance to salt (NaCl) stress. In addition, they found that depletion of β-COP caused an increased length of Golgi stacks, which in some cases seemed to be the consequence of lateral fusion between Golgi stacks. This suggests that β-COP function is required for maintaining the structure of the plant Golgi apparatus.

Kaiser and Scheuring discuss the contribution of the plant vacuole to cell elongation. Recent data revealed that the receptor-like kinase FERONIA together with extracellular leucine-rich repeat extensins sense cell wall properties such as loosening and subsequently impact on the intracellular expansion of the vacuole. Therefore, the authors deliberate on the possible connection between cell wall status and vacuolar morphology. In particular, they offer their view on the question whether vacuolar size is dictated by cell size or vice versa.

The conserved exocyst complex plays multiple functions in plant growth, cell wall synthesis, immunity and hormone signaling. In many plant species, there are multiple EXO70 genes and their functional redundancy and specialization has been an important question in understanding the molecular mechanisms of exocyst complex function. The manuscript by Pečenková et al. aims to find any functional redundancy and specialization among selected EXO70 isoforms during plant response to biotic stress. The authors analyse existing transcriptome data with focus on response of individual paralogs to biotic stresses. In addition to data mining, the transcripts of selected EXO70 genes in exo70A1 background were detected to see which other EXO70s compensate for the absence of EXO70A1. The authors then analyse various single mutants, combinations of double mutants, and a triple mutant in their response to pathogens using a root hair growth stimulation assay and a flooding assay. It was revealed that different EXO70 isoforms have both redundant and specialized functions and that different EXO70 mutants have compensatory effects. Especially interesting was the *exo70B1 exo70B2* double mutant and therefore the structure of EXO70B1 and EXO70B2 was modeled to see whether the C-terminal lipid-binding motif might contribute to the diverged function of the two proteins.

In the review article by Schwihla and Korbei, the authors provide a nice and timely overview of the latest knowledge on key steps of endocytic degradation of plasma membrane proteins in plants—from clathrin-mediated endocytosis, adaptor proteins, small GTPases to the ESCRT machinery. The authors also highlight the role of posttranslational modifications in these processes. Similar to what has been reported in other eukaryotes, phosphorylation and ubiquitination are important protein modifications for the correct determination of protein fate. Recent findings suggest that although many basic mechanisms are conserved in the plant endosomal degradation pathway when compared to animals, there are also a significant number of molecular processes relying on plant-specific regulators. Efforts are being made to understand the crosstalk between various intracellular trafficking pathways and the implication of endocytic degradation in plants.

The regulation of autophagy and its physiological function is a topic that fascinates many researchers across the broad field of plant biology. The review from the Boycheva Woltering and Isono summarizes molecules that were reported to affect autophagic activity in plants. These factors include metal ions, sugar, amino acids, radicals to phytohormones and, in most cases, they affect directly or indirectly the activity of the TOR kinase complex enabling the plant to adapt its autophagic activity to the environmental conditions. The authors also discuss interesting differences in ATG8 homologs. In contrast to ATG8a-e, which require processing at the C-terminus by the protease ATG4, ATG8h, and ATG8i have an exposed glycine at the C-terminus and theoretically do not require the activity of ATG4 before it can be conjugated. This seemingly “cleavage-free” ATG8 variants are found in all examined species and are expressed genes in Arabidopsis. Whether and what impact this differences in ATG8 have on their biochemical and biological characteristics is an emerging research question.

Autophagy has been primarily associated with nutrient recycling in plants, however, as recent studies have shown, its physiological implication is broad. The review by Norizuki et al. compares male reproductive process in mammals and plants. Though there are fundamental differences, in both systems, cytosolic contents are removed directly or indirectly by autophagy. In Mosses, an increased number of autophagosomes is observed during spermiogenesis, and defect in the autophagy pathway in *Physcomitrella patens* causes morphological abnormalities of the spermatozoid. In angiosperms, autophagy seems to be dispensable for fertility *per se*, as *atg* mutants in *Arabidopsis* and Maize are fertile. In rice, however, autophagy mutants show decreased fertility. The review cast light on the yet poorly understood molecular regulation of autophagy during developmental processes and provide an excellent overview on the involvement of autophagy in gametogenesis.

True to his undergrad training as an engineer, Daniel Van Damme has a recurrent record of introducing novel technologies for research. On this occasion, his group reports on an elegant method for knocking down gene activity through nanobody-based delocalization of tagged proteins (Winkler et al.). As a proof of concept, they express an anti-GFP nanobody under the control of the PIN2 promoter and target it to the mitochondrial outer membrane. Crossing this construct into a null mutant of the TPLATE complex subunit TML complemented with TML-GFP results in mitochondrial-sequestration of TML-GFP and an ensuing reduction in endocytic fluxes in root epidermal and cortex cells, without affecting root growth or plant viability. These results demonstrate the power of this technology to achieve gene inactivation in a tissue-specific manner, or conditionally if inducible promoters are used, which is particularly relevant for functional studies of essential genes.

Another interesting technology, serial block face scanning electron microscopy (SBF-SEM), is presented in the perspective article from the Stoger lab. This technique allows 3D ultrastructural analysis of large sample volumes and in this report it is used to image developing maize seeds (Arcalís et al.). Their 3D models reveal that endoplasmic reticulum in the endosperm consists primarily of large sheets and that protein bodies bud from central areas of those sheets rather than from zones of higher curvature such as tubules or edges. The use of SBF-SEM is relatively new in plants, and this work is a good example of how it can be used to resolve open questions in plant cell biology.

## Author Contributions

All authors listed have made a substantial, direct and intellectual contribution to the work, and approved it for publication.

## Conflict of Interest

The authors declare that the research was conducted in the absence of any commercial or financial relationships that could be construed as a potential conflict of interest.

